# Supplementary material for: Chemical patterns of colony membership and mother-offspring similarity in Antarctic fur seals are reproducible
Source: PeerJ. 2020 Oct 19;8:e10131. doi: 10.7717/peerj.10131 (PMC7580581; doi:10.7717/peerj.10131)
Supplement: Supplemental Information 1 [file peerj-08-10131-s001.docx]

| **(a) PERMANOVAs of animals from different colonies** | ***F*** | ***R*^2^** | ***p*-value** | **Corrected *p*-value** |
| --- | --- | --- | --- | --- |
| SSB mothers versus FWB mothers | 4.27 | 0.082 | <0.0001 | <0.001 |
| SSB mothers versus FWB pups | 4.22 | 0.081 | <0.0001 | <0.001 |
| SSB pups versus FWB mothers | 6.62 | 0.119 | <0.00001 | <0.0001 |
| SSB pup versus FWB pups | 6.20 | 0.112 | <0.00001 | <0.0001 |
| **(b) PERMANOVAs of animals from the same colonies** | ***F*** | ***R*^2^** | ***p*-value** | **Corrected *p*-value** |
| SSB mothers versus SSB pups | 2.42 | 0.047 | 0.013 | 0.074 |
| FWB mothers versus FWB pups | 2.21 | 0.044 | 0.019 | 0.114 |
