# Supplementary material for: Chemical patterns of colony membership and mother-offspring similarity in Antarctic fur seals are reproducible
Source: PeerJ. 2020 Oct 19;8:e10131. doi: 10.7717/peerj.10131 (PMC7580581; doi:10.7717/peerj.10131)
Supplement: Supplemental Information 2 [file peerj-08-10131-s002.docx]

| **Test for homogeneity of variance** | ***F*** | ***p*-value** | **Corrected *p*-value** |
| --- | --- | --- | --- |
| SSB mothers versus SSB pups | 0.10 | 0.750 | 1.000 |
| SSB mothers versus FWB mothers | 3.52 | 0.067 | 0.467 |
| SSB mothers versus FWB pups | 0.95 | 0.334 | 1.000 |
| SSB pups versus FWB mothers | 4.58 | 0.037 | 0.261 |
| SSB pup versus FWB pups | 1.63 | 0.208 | 1.000 |
| FWB mothers versus FWB pups | 0.85 | 0.362 | 1.000 |
| All pairs combined | 1.91 | 0.134 | 0.935 |
