# Supplementary figures and images for: Chemical patterns of colony membership and mother-offspring similarity in Antarctic fur seals are reproducible
Source: PeerJ. 2020 Oct 19;8:e10131. doi: 10.7717/peerj.10131 (PMC7580581; doi:10.7717/peerj.10131)

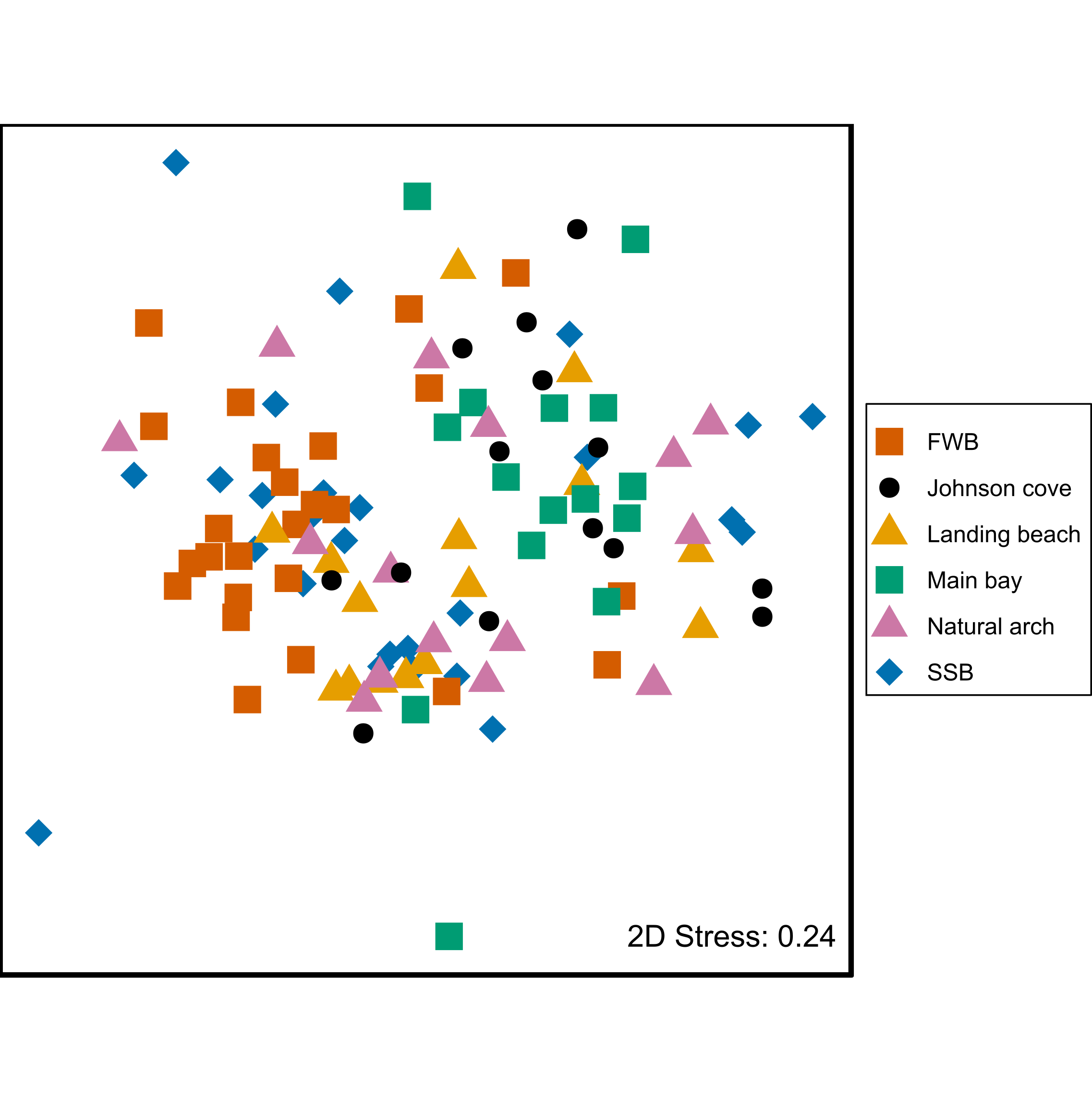

Supplement: Supplemental Information 3 — NDMS was performed using Bray–Curtis similarity values calculated from log(x+1) transformed chemical abundance data. The scales of the two axes are arbitrary and the closer two points appear in the plot, the more similar they are chemically. Individual data points are color-coded by colony. [file peerj-08-10131-s003.png]
