# Supplementary material for: Chemical patterns of colony membership and mother-offspring similarity in Antarctic fur seals are reproducible
Source: PeerJ. 2020 Oct 19;8:e10131. doi: 10.7717/peerj.10131 (PMC7580581; doi:10.7717/peerj.10131)
Supplement: Supplemental Information 4 [file peerj-08-10131-s004.pdf]

Chemical patterns of colony membership and mother-offspring similarity in Antarctic fur seals are reproducible - R Code

Jonas Tebbe, Emily Humble, Martin A. Stoffel, Lisa Johanna Tewes, Caroline Müller, Jaume Forcada, Barbara Caspers & Joseph Ivan Hoffman

July 2020

Used packages

Install with "install.packages". After installation, packages can be called with 'library' oder 'require'

```
library(GCalignR)
library(vegan)
library(readr)
library(ggplot2)
library(ggbeeswarm)
library(tidyverse)
library(pairwiseAdonis)
```

Alignment procedure

```
# Alignment protocol to align a data frame with GCMS peaks for
# mother-pup pairs and pure pup colonies. For comparability, the
# six colonies only include pure pup data and thus, need a different
# alignment protocol, as algorithm properties are inherited within
# the function but not across functions callings. In consequence,
# alignment is more accurate for a set data subset.


load("RData/objects/seal_raw_dfs.Rdata")

# index correct MP & pup_colony subset
index_MP <- c(1:61,72:111)
index_pupcols <- c(51:160)

# Aligning mom-pup colonies
mom_pup_aligned <- align_chromatograms(
  data = all_dfs[index_MP], # input data
  rt_col_name = "RT", # retention time variable name
  rt_cutoff_low = 15, # remove peaks below 15 Minutes
  rt_cutoff_high = 54.7, # remove peaks exceeding 54.7 Minutes
  reference = "P13", # sample with highest overall peak number
  max_linear_shift = 0.05, # Premise 1: max. shift for linear corrections
  max_diff_peak2mean = 0.08, # Premise 2: max. distance of a peak to the mean across samples
  min_diff_peak2peak = 0.03, # Premise 3: min. expected distance between peaks
  delete_single_peak = T, # delete peaks that are present in just one sample
```

```r
  write_output = NULL) # add variable names to write aligned data to text files

# Aligning six pup colonies
pup_colonies_aligned <- align_chromatograms(
  data = all_dfs[index_pupcols], # input data
  rt_col_name = "RT", # retention time variable name
  rt_cutoff_low = 15, # remove peaks below 15 Minutes
  rt_cutoff_high = 54.7, # remove peaks exceeding 54.7 Minutes
  reference = "P13",# sample with highest overall peak number
  max_linear_shift = 0.05, # Premise 1: max. shift for linear corrections
  max_diff_peak2mean = 0.08, # Premise 2: max. distance of a peak to the mean across samples
  min_diff_peak2peak = 0.03, # Premise 3: min. expected distance between peaks
  delete_single_peak = T, # delete peaks that are present in just one sample
  write_output = NULL) # add variable names to write aligned data to text files
```

Alignment and preliminary data properties

```r
## Load and view GCalignR alignment
## objects for GCMS scent data in two and
## six breeding beaches
load("RData/objects/mom_pup_alignment_GCalignR.RData")
mom_pup_aligned
```

```
## Summary of Peak Alignment running align_chromatograms
## Input: all_dfs[index2]
## Start:  2019-05-21 11:41:08  Finished:  2019-05-21 11:44:23
##
## Call:
##   GCalignR::align_chromatograms(data=[, data=all_dfs, data=index2, rt_col_name=RT,
##   rt_cutoff_low=15, rt_cutoff_high=54.7, reference=P13, max_linear_shift=0.05,
##   max_diff_peak2mean=0.08, min_diff_peak2peak=0.03, delete_single_peak=T,
##   sep=\t, ...=)
##
## Summary of scored substances:
##    total singular retained
##      157       39      118
##
## In total 157 substances were identified among all samples. 39 substances were
##   present in just one single sample and were removed. 118 substances are retained
##   after all filtering steps.
##
## Sample overview:
##   The following 101 samples were aligned to the reference 'P13':
##   M01, M02, M03, M04, M05, M06, M07, M08, M09, M10, M11, M12, M13, M14, M15, M16,
##   M17, M18, M19, M20, M21, M22, M23, M24, M25, M26, M27, M28, M29, M30, M31, M32,
##   M33, M34, M35, M36, M37, M38, M39, M40, M41, M42, M43, M44, M45, M46, M47, M48,
##   M49, M50, P01, P02, P03, P04, P05, P06, P07, P07b, P08, P09, P10, P11, P12, P13,
##   P14, P15, P16, P17, P18, P19, P20, P21, P22, P23, P24, P25, P26, P27, P28, P29,
##   P30, P31, P32, P33, P34, P35, P36, P37, P38, P39, P40, P41, P42, P43, P44, P45,
##   P46, P47, P48, P49, P50
##
## For further details type:
##   'gc_heatmap(x)' to retrieve heatmaps
```

```
##   'plot(x)' to retrieve further diagnostic plots
```

```r
load("RData/objects/pup_colonies_alignment_GCalignR.RData")
pup_colonies_aligned
```

```
## Summary of Peak Alignment running align_chromatograms
## Input: all_dfs[index4]
## Start:  2019-05-23 11:36:39  Finished:  2019-05-23 11:40:15
##
## Call:
##   GCalignR::align_chromatograms(data=[, data=all_dfs, data=index4, rt_col_name=RT,
##   rt_cutoff_low=15, rt_cutoff_high=54.7, reference=P13, max_linear_shift=0.05,
##   max_diff_peak2mean=0.08, min_diff_peak2peak=0.03, delete_single_peak=T,
##   sep=\t, ...=)
##
## Summary of scored substances:
##    total singular retained
##      143       28      115
##
## In total 143 substances were identified among all samples. 28 substances were
##   present in just one single sample and were removed. 115 substances are retained
##   after all filtering steps.
##
## Sample overview:
##   The following 110 samples were aligned to the reference 'P13':
##   P01, P02, P03, P04, P05, P06, P07, P07b, P08, P09, P10, P100, P101, P102, P103,
##   P104, P105, P106, P107, P108, P109, P11, P12, P13, P14, P15, P16, P17, P18, P19,
##   P20, P21, P22, P23, P24, P25, P26, P27, P28, P29, P30, P31, P32, P33, P34, P35,
##   P36, P37, P38, P39, P40, P41, P42, P43, P44, P45, P46, P47, P48, P49, P50, P51,
##   P52, P53, P54, P55, P56, P57, P58, P59, P60, P61, P62, P63, P64, P65, P66, P67,
##   P68, P69, P70, P71, P72, P73, P74, P75, P76, P77, P78, P79, P80, P81, P82, P83,
##   P84, P85, P86, P87, P88, P89, P90, P91, P92, P93, P94, P95, P96, P97, P98, P99
##
## For further details type:
##   'gc_heatmap(x)' to retrieve heatmaps
##   'plot(x)' to retrieve further diagnostic plots
```

```r
## Load raw information for all samples
## containing raw peaks and calculate mean
## peak number
load("RData/objects/seal_raw_dfs.Rdata")

individual_peak_number <- NULL
for (i in 1:length(seal_dfs.list)) {
    individual_peak_number[i] <- length(seal_dfs.list[[i]]$RT)
}

mean_ind_peaks <- mean(individual_peak_number)
sd_ind_peaks <- sd(individual_peak_number)
cat("\n", "\n", "Mean peaks:", as.character(mean_ind_peaks),
    "\n", "Peak SD:", as.character(sd_ind_peaks))
```

```
##
##
##  Mean peaks: 34.175
```

```
##  Peak SD: 10.8445563540296
```

NMDS scaling of mother-pup alignment data

```r
load("RData/objects/mom_pup_alignment_GCalignR.RData")

scent_factors_raw <- read_delim("documents/metadata_seal_scent.txt",
    "\t", escape_double = FALSE, trim_ws = TRUE)
scent_factors_raw <- as.data.frame(scent_factors_raw[-c(194:209),
    ])

# set sample names as row names, ensure
# there are no duplicates
scent_factors <- scent_factors_raw[, -1]
rownames(scent_factors) <- scent_factors_raw[,
    1]

## check for empty samples, i.e. no peaks
x <- apply(mom_pup_aligned$aligned$RT, 2,
    sum)
x <- which(x == 0)

## normalise area and return a data frame
scent <- norm_peaks(mom_pup_aligned, conc_col_name = "Area",
    rt_col_name = "RT", out = "data.frame")
## common transformation for abundance
## data to reduce the extent of
## mean-variance trends
scent <- log(scent + 1)

## subset scent_factors
scent_factors <- scent_factors[rownames(scent_factors) %in%
    rownames(scent), ]
scent <- scent[rownames(scent) %in% rownames(scent_factors),
    ]

## keep order of rows consistent
scent <- scent[match(rownames(scent_factors),
    rownames(scent)), ]

## get number of compounds for each
## individual sample after alignment
num_comp <- as.vector(apply(scent, 1, function(x) length(x[x >
    0])))

## bray-curtis similarity
scent_nmds.obj <- vegan::metaMDS(comm = scent,
    k = 2, try = 999, trymax = 9999, distance = "bray")

scent_nmds <- as.data.frame(scent_nmds.obj[["points"]])

scent_nmds <- cbind(scent_nmds, age = scent_factors[["age"]],
    tissue_tag = scent_factors[["tissue_tag"]],
```

```r
    colony = scent_factors[["colony"]], family = as.factor(scent_factors[["family"]]),
    clr = as.factor(scent_factors[["clr"]]),
    shp = as.factor(scent_factors[["shp"]]),
    gcms = as.factor(scent_factors[["gcms_run"]]),
    peak_res = as.factor(scent_factors[["peak_res"]]),
    sample_qlty = as.factor(scent_factors[["sample_qlty"]]),
    vialdate = as.factor(scent_factors[["gcms_vialdate"]]),
    captured = as.factor(scent_factors[["capture_date"]]),
    sex = scent_factors[["sex"]], num_comp = num_comp)
scent_nmds <- scent_nmds %>% mutate(BeachAge = str_c(colony,
    age, sep = "_"))
# creates & adds new variable BeachAge
# and simplifies plotting
```

Colony and family membership in SSB and FWB mom-pup pairs

```r
load("RData/objects/mom_pup_nmds_scaling.RData")

## colony membership plot
mp_colony_gg <- ggplot(data = scent_nmds) +
    geom_point(size = 4.5, aes(MDS1, MDS2,
        color = BeachAge, shape = BeachAge)) +
    scale_shape_manual(values = c(19, 1,
        19, 1), labels = c("FWB mothers ",
        "FWB pups ", "SSB mothers ", "SSB pups ")) +
    scale_color_manual(values = c("#D55E00",
        "#D55E00", "#56B4E9", "#56B4E9"),
        labels = c("FWB mothers ", "FWB pups ",
            "SSB mothers ", "SSB pups ")) +
    theme_void() + ylim(-0.75, 1.1) + annotate("text",
    x = 0.64, y = 1.1, label = "A", size = 5) +
    annotate("text", x = 0.47, y = -0.74,
        label = "2D Stress: 0.23", size = 4) +
    theme(panel.background = element_rect(colour = "black",
        size = 1, fill = NA), aspect.ratio = 1,
        legend.position = "none", legend.title = element_blank(),
        legend.background = element_rect(size = 0.3,
            linetype = "solid", color = "black"))
# call colony membership plot
mp_colony_gg
```

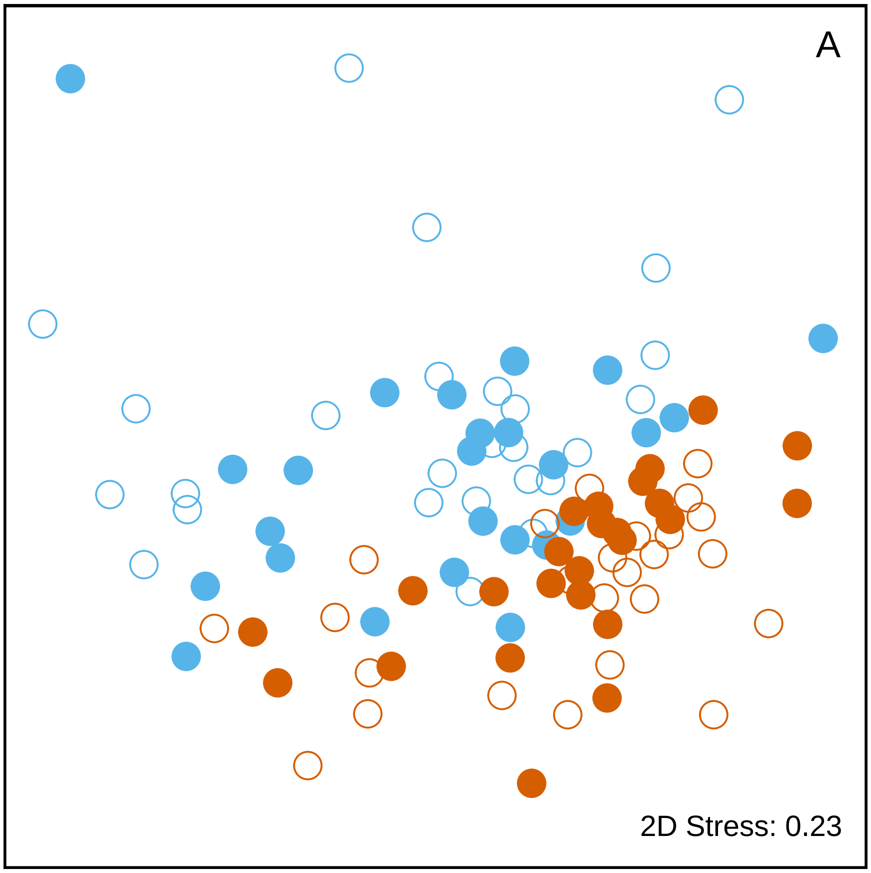


```
## mother-offspring similarity plot create
## color palette for the plot
clr <- c("#D55E00", "red", "#56B4E9", "#009E73",
    "#000000", "#CC79A7")

# assign pch values for plotting
shp <- c(0, 1, 2, 7, 10, 5, 6, 18, 16, 17,
    15)

# create unique color-pch pairs
color_shape_pairs <- crossing(clr, shp)

# randomly sample 50 unique pairs (sample
# without replacement)
set.seed(123)  # always get same pairs in a run
color_shape_pairs <- color_shape_pairs[sample(nrow(color_shape_pairs),
    50), ]

# assign new dataframes to transform
# scent_nmds$clr & shp with the unique
# values we created
color_shape_pairs_plot <- rbind(color_shape_pairs[1:25,
    ], color_shape_pairs[1:7, ], color_shape_pairs[7,
    ], color_shape_pairs[8:25, ], color_shape_pairs[26:50,
    ], color_shape_pairs[26:50, ])
scent_nmds$clr <- as.factor(color_shape_pairs_plot$clr)
```

```
scent_nmds$shp <- as.factor(color_shape_pairs_plot$shp)

# call family plot
mp_family_gg <- ggplot(data = scent_nmds,
    aes(MDS1, MDS2, color = clr, shape = shp)) +
    geom_point(size = 4.5) + # geom_line() +
scale_shape_manual(values = as.numeric(levels(scent_nmds$shp))) +
    theme_void() + ylim(-0.75, 1.1) + scale_color_manual(values = levels(scent_nmds$clr)) +
    annotate("text", x = 0.64, y = 1.1, label = "B",
        size = 5) + annotate("text", x = 0.48,
    y = -0.74, label = "2D Stress: 0.23",
    size = 4) + theme(panel.background = element_rect(colour = "black",
    size = 1, fill = NA), aspect.ratio = 1,
    legend.position = "none")
mp_family_gg
```

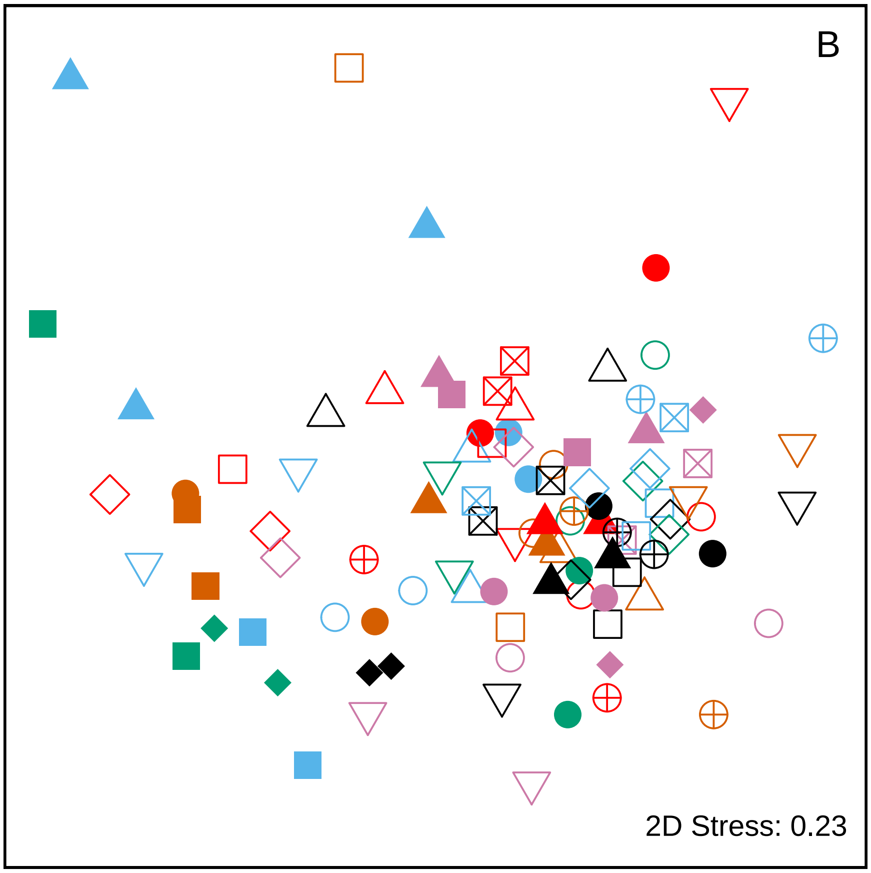

PERMANOVA for mother-pup similiarity and colony membership

```
# set seed to reproduce the same outcome
# (can vary due to different
# permutations!)
set.seed(123)

# set counter for while loop
perm_count <- c(99)
```

```r
# iterate different significance levels
# with a while-loop end while-loop after
# the run for 99999 permutations
while (perm_count <= 99999) {
    permanova_result_MP <- adonis(scent ~
        age + colony + colony:family, data = scent_factors,
        method = "bray", permutations = perm_count)
    print(permanova_result_MP)

    perm_count <- (perm_count * 10) + 9  # ends while-iteration after it increases to 999999
}  #while
```

```
##
## Call:
## adonis(formula = scent ~ age + colony + colony:family, data = scent_factors,      permutations = per
##
## Permutation: free
## Number of permutations: 99
##
## Terms added sequentially (first to last)
##
##                 Df SumsOfSqs MeanSqs F.Model      R2 Pr(>F)
## age              1    0.3217 0.32170  2.6896 0.02253   0.02 *
## colony           1    1.0847 1.08475  9.0692 0.07599   0.01 **
## colony:family    2    1.3870 0.69351  5.7982 0.09716   0.01 **
## Residuals       96   11.4823 0.11961         0.80432
## Total          100   14.2758                 1.00000
## ---
## Signif. codes:  0 '***' 0.001 '**' 0.01 '*' 0.05 '.' 0.1 ' ' 1
##
## Call:
## adonis(formula = scent ~ age + colony + colony:family, data = scent_factors,      permutations = per
##
## Permutation: free
## Number of permutations: 999
##
## Terms added sequentially (first to last)
##
##                 Df SumsOfSqs MeanSqs F.Model      R2 Pr(>F)
## age              1    0.3217 0.32170  2.6896 0.02253  0.011 *
## colony           1    1.0847 1.08475  9.0692 0.07599  0.001 ***
## colony:family    2    1.3870 0.69351  5.7982 0.09716  0.001 ***
## Residuals       96   11.4823 0.11961         0.80432
## Total          100   14.2758                 1.00000
## ---
## Signif. codes:  0 '***' 0.001 '**' 0.01 '*' 0.05 '.' 0.1 ' ' 1
##
## Call:
## adonis(formula = scent ~ age + colony + colony:family, data = scent_factors,      permutations = per
##
## Permutation: free
## Number of permutations: 9999
##
```

```
## Terms added sequentially (first to last)
##
##                Df SumsOfSqs MeanSqs F.Model      R2 Pr(>F)
## age             1    0.3217 0.32170  2.6896 0.02253 0.0045 **
## colony          1    1.0847 1.08475  9.0692 0.07599 0.0001 ***
## colony:family   2    1.3870 0.69351  5.7982 0.09716 0.0001 ***
## Residuals      96   11.4823 0.11961         0.80432
## Total         100   14.2758                 1.00000
## ---
## Signif. codes:  0 '***' 0.001 '**' 0.01 '*' 0.05 '.' 0.1 ' ' 1
##
## Call:
## adonis(formula = scent ~ age + colony + colony:family, data = scent_factors,      permutations = per
##
## Permutation: free
## Number of permutations: 99999
##
## Terms added sequentially (first to last)
##
##                Df SumsOfSqs MeanSqs F.Model      R2  Pr(>F)
## age             1    0.3217 0.32170  2.6896 0.02253 0.00403 **
## colony          1    1.0847 1.08475  9.0692 0.07599   1e-05 ***
## colony:family   2    1.3870 0.69351  5.7982 0.09716   1e-05 ***
## Residuals      96   11.4823 0.11961         0.80432
## Total         100   14.2758                 1.00000
## ---
## Signif. codes:  0 '***' 0.001 '**' 0.01 '*' 0.05 '.' 0.1 ' ' 1
```

Post-hoc betadisper and pairwise comparisons for mother-pup pair PERMANOVA results

```
# test for group dispersal for different
# colonies
mod_colony <- betadisper(vegdist(scent),
    scent_factors$colony, type = "median")
anova(mod_colony)
```

```
## Analysis of Variance Table
##
## Response: Distances
##           Df Sum Sq  Mean Sq F value  Pr(>F)
## Groups     1 0.0442 0.044201   5.136 0.02561 *
## Residuals 99 0.8520 0.008606
## ---
## Signif. codes:  0 '***' 0.001 '**' 0.01 '*' 0.05 '.' 0.1 ' ' 1
```

```
# for different ages (mom vs pup)
mod_age <- betadisper(vegdist(scent), scent_factors$age,
    type = "median")
anova(mod_age)
```

```
## Analysis of Variance Table
##
## Response: Distances
##           Df  Sum Sq   Mean Sq F value Pr(>F)
## Groups     1 0.01305 0.0130477  1.4726 0.2278
## Residuals 99 0.87718 0.0088604
```

```r
# for different combinations of age and
# colony identity
mod_BeachAge <- betadisper(vegdist(scent),
    scent_nmds$BeachAge, type = "median")
anova(mod_BeachAge)
```

```
## Analysis of Variance Table
##
## Response: Distances
##           Df  Sum Sq   Mean Sq F value Pr(>F)
## Groups     3 0.04845 0.0161488  1.9067 0.1336
## Residuals 97 0.82153 0.0084694
```

Pairwise comparison between all possible combinations of mother-pup pairs based on colony identity and age

```r
set.seed(123)  # for comparability as results do not change when compared to manuscript
pairwiseAdonis::pairwise.adonis(scent, scent_nmds$BeachAge,
    perm = 99999)
```

```
##            pairs Df SumsOfSqs  F.Model         R2 p.value p.adjusted sig
## 1 SSB_1 vs SSB_2  1 0.3515433 2.415725 0.04698416 0.01227    0.07362
## 2 SSB_1 vs FWB_1  1 0.5291662 4.272400 0.08173337 0.00004    0.00024  **
## 3 SSB_1 vs FWB_2  1 0.5542266 4.218847 0.08079165 0.00003    0.00018  **
## 4 SSB_2 vs FWB_1  1 0.8481620 6.618475 0.11899778 0.00001    0.00006 ***
## 5 SSB_2 vs FWB_2  1 0.8397841 6.197227 0.11227424 0.00001    0.00006 ***
## 6 FWB_1 vs FWB_2  1 0.2514355 2.212658 0.04406574 0.01906    0.11436
```

```r
# Pairwise group dispersal tests

# scent, scent_factors and scent_nmds are
# all data.frames the have the same row
# setup.  Meaning: Each row respresenting
# an individual is identical in each
# data.frame.  Thus, we can index pairs
# based on scent_nmds$BeachAge manually.

pairwise_betadisper1 <- betadisper(vegdist(scent[scent_nmds$BeachAge ==
    "SSB_1" | scent_nmds$BeachAge == "SSB_2",
    ]), scent_nmds$BeachAge[scent_nmds$BeachAge ==
    "SSB_1" | scent_nmds$BeachAge == "SSB_2"],
    type = "median")
anova(pairwise_betadisper1)
```

```
## Analysis of Variance Table
##
## Response: Distances
##           Df  Sum Sq   Mean Sq F value Pr(>F)
## Groups     1 0.00104 0.0010419  0.1024 0.7504
## Residuals 49 0.49872 0.0101780
```

```r
pairwise_betadisper2 <- betadisper(vegdist(scent[scent_nmds$BeachAge ==
    "SSB_1" | scent_nmds$BeachAge == "FWB_1",
    ]), scent_nmds$BeachAge[scent_nmds$BeachAge ==
    "SSB_1" | scent_nmds$BeachAge == "FWB_1"],
    type = "median")
anova(pairwise_betadisper2)
```

```
## Analysis of Variance Table
##
## Response: Distances
##           Df  Sum Sq   Mean Sq F value  Pr(>F)
## Groups     1 0.02731 0.0273093  3.5184 0.06678 .
## Residuals 48 0.37256 0.0077618
## ---
## Signif. codes:  0 '***' 0.001 '**' 0.01 '*' 0.05 '.' 0.1 ' ' 1
```

```
pairwise_betadisper3 <- betadisper(vegdist(scent[scent_nmds$BeachAge ==
    "SSB_1" | scent_nmds$BeachAge == "FWB_2",
    ]), scent_nmds$BeachAge[scent_nmds$BeachAge ==
    "SSB_1" | scent_nmds$BeachAge == "FWB_2"],
    type = "median")
anova(pairwise_betadisper3)
```

```
## Analysis of Variance Table
##
## Response: Distances
##           Df  Sum Sq   Mean Sq F value Pr(>F)
## Groups     1 0.00796 0.0079639  0.9519 0.3341
## Residuals 48 0.40159 0.0083665
```

```
pairwise_betadisper4 <- betadisper(vegdist(scent[scent_nmds$BeachAge ==
    "SSB_2" | scent_nmds$BeachAge == "FWB_1",
    ]), scent_nmds$BeachAge[scent_nmds$BeachAge ==
    "SSB_2" | scent_nmds$BeachAge == "FWB_1"],
    type = "median")
anova(pairwise_betadisper4)
```

```
## Analysis of Variance Table
##
## Response: Distances
##           Df  Sum Sq  Mean Sq F value  Pr(>F)
## Groups     1 0.03973 0.039734  4.5784 0.03738 *
## Residuals 49 0.42525 0.008679
## ---
## Signif. codes:  0 '***' 0.001 '**' 0.01 '*' 0.05 '.' 0.1 ' ' 1
```

```
pairwise_betadisper5 <- betadisper(vegdist(scent[scent_nmds$BeachAge ==
    "SSB_2" | scent_nmds$BeachAge == "FWB_2",
    ]), scent_nmds$BeachAge[scent_nmds$BeachAge ==
    "SSB_2" | scent_nmds$BeachAge == "FWB_2"],
    type = "median")
anova(pairwise_betadisper5)
```

```
## Analysis of Variance Table
##
## Response: Distances
##           Df  Sum Sq  Mean Sq F value Pr(>F)
## Groups     1 0.01502 0.015016  1.6302 0.2077
## Residuals 49 0.45134 0.009211
```

```
pairwise_betadisper6 <- betadisper(vegdist(scent[scent_nmds$BeachAge ==
    "FWB_1" | scent_nmds$BeachAge == "FWB_2",
    ]), scent_nmds$BeachAge[scent_nmds$BeachAge ==
```

```r
    "FWB_1" | scent_nmds$BeachAge == "FWB_2"],
    type = "median")
anova(pairwise_betadisper6)
```

```
## Analysis of Variance Table
##
## Response: Distances
##           Df  Sum Sq   Mean Sq F value Pr(>F)
## Groups     1 0.00579 0.0057933  0.8476 0.3618
## Residuals 48 0.32807 0.0068347
```

Bonferroni correction for pairwise betadisper

```r
# get p-values for betadisper (same
# order)
betadisper_sig_values <- c(0.1336, 0.7504,
    0.06678, 0.3341, 0.03738, 0.2077, 0.3618)

# correct p-values based on Bonferroni
corrected_betadisper_sig_values <- p.adjust(betadisper_sig_values,
    method = "bonferroni")
corrected_betadisper_sig_values
```

```
## [1] 0.93520 1.00000 0.46746 1.00000 0.26166 1.00000 1.00000
```

NMDS scaling and colony membership in six pup colonies

```r
load("RData/objects/pup_colonies_alignment_GCalignR.RData")
scent_factors_raw <- read_delim("documents/metadata_seal_scent.txt",
    "\t", escape_double = FALSE, trim_ws = TRUE)
scent_factors_raw <- as.data.frame(scent_factors_raw[-c(194:209),
    ])

# set sample names as row names, ensure
# there are no duplicates
scent_factors <- scent_factors_raw[, -1]
rownames(scent_factors) <- scent_factors_raw[,
    1]

## check for empty samples, i.e. no peaks
x <- apply(pup_colonies_aligned$aligned$RT,
    2, sum)
x <- which(x == 0)

## normalise area and return a data frame
scent <- norm_peaks(pup_colonies_aligned,
    conc_col_name = "Area", rt_col_name = "RT",
    out = "data.frame")
## common transformation for abundance
## data to reduce the extent of
## mean-variance trends
scent <- log(scent + 1)

## subset scent_factors
```

```r
scent_factors <- scent_factors[rownames(scent_factors) %in%
    rownames(scent), ]
scent <- scent[rownames(scent) %in% rownames(scent_factors),
    ]

## keep order of rows consistent
scent <- scent[match(rownames(scent_factors),
    rownames(scent)), ]

## get number of compounds for each
## individual sample after alignment
num_comp <- as.vector(apply(scent, 1, function(x) length(x[x >
    0])))

## bray-curtis similarity
scent_nmds.obj <- metaMDS(comm = scent, k = 2,
    try = 999, trymax = 9999, distance = "bray")
## MDS outcome evaluated with PCA for
## factor colony in metadata table for
## individuals
scent_nmds <- with(scent_factors, MDSrotate(scent_nmds.obj,
    colony))

## get x and y coordinates
scent_nmds <- as.data.frame(scent_nmds[["points"]])

## add the colony as a factor to each
## sample
scent_nmds <- cbind(scent_nmds, age = scent_factors[["age"]],
    tissue_tag = scent_factors[["tissue_tag"]],
    colony = scent_factors[["colony"]], family = as.factor(scent_factors[["family"]]),
    clr = as.factor(scent_factors[["clr"]]),
    shp = as.factor(scent_factors[["shp"]]),
    gcms = as.factor(scent_factors[["gcms_run"]]),
    peak_res = as.factor(scent_factors[["peak_res"]]),
    sample_qlty = as.factor(scent_factors[["sample_qlty"]]),
    vialdate = as.factor(scent_factors[["gcms_vialdate"]]),
    captured = as.factor(scent_factors[["capture_date"]]),
    sex = scent_factors[["sex"]], num_comp = num_comp)
# creates & adds new variable BeachAge
scent_nmds <- scent_nmds %>% mutate(BeachAge = str_c(colony,
    age, sep = "_"))
```

Colony membership plot for six pup colonies (Supplementary figure)

```r
load("RData/objects/pup_colonies_nmds_scaling.RData")

pup_colony_gg <- ggplot(data = scent_nmds, aes(MDS1, MDS2, color = colony, shape = colony)) +
  geom_point(size = 4.5) +
  scale_shape_manual(values = c(15,20,17,15,17,18),
                     labels = c("FWB", "Johnson cove", "Landing beach", "Main bay", "Natural arch", "SSI
  scale_color_manual(values = c("#D55E00", "#000000", "#E69F00", "#009E73", "#CC79A7", "#0072B2"),
                     labels = c("FWB", "Johnson cove", "Landing beach", "Main bay", "Natural arch", "SSI
  theme_void() +
```

```
  annotate("text", x = 0.6, y = -0.94, label = "2D Stress: 0.24", size = 4) +
  theme(panel.background = element_rect(colour = "black", size = 1, fill = NA),
        aspect.ratio = 1,
        legend.position = "right", #c(0.1,0.87),
        legend.title = element_blank(),
        # legend.key.size = unit(0.5, "cm"),
        # legend.key.width = unit(0.5, "cm"),
        legend.background = element_rect(size = 0.3, linetype = "solid", color = "black"))

pup_colony_gg
```

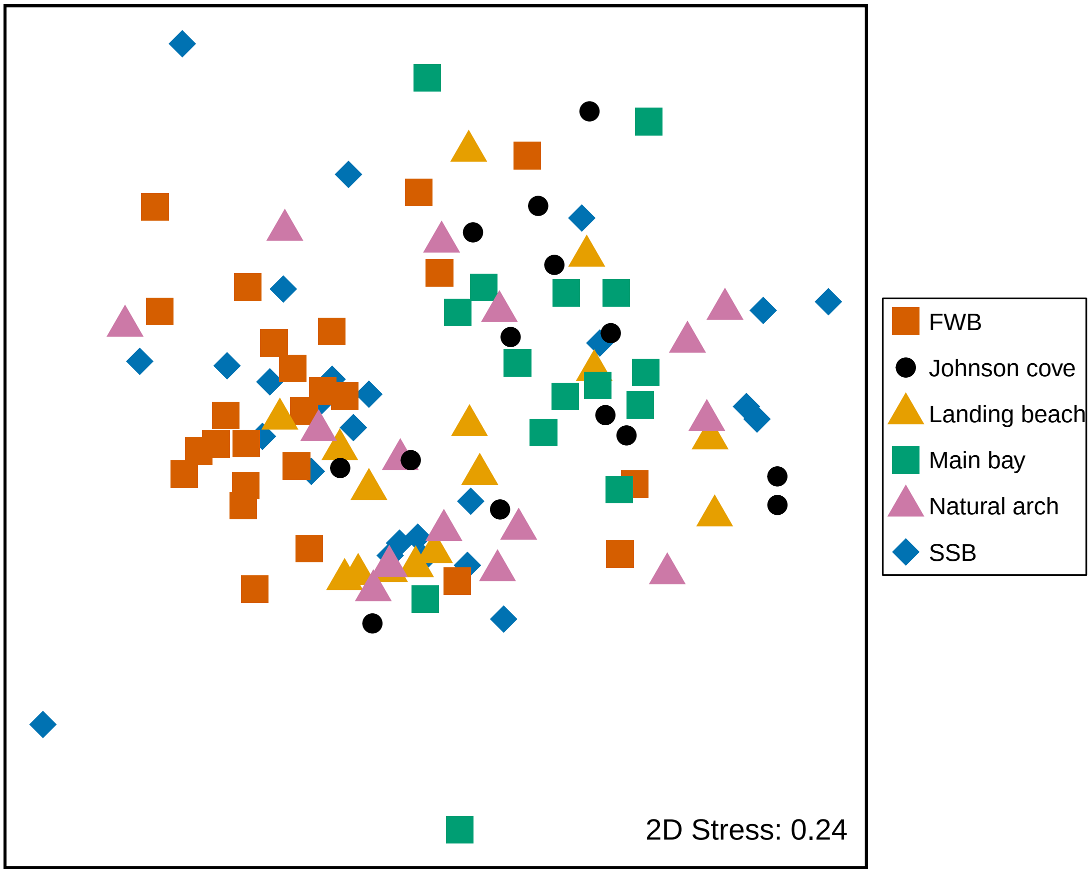

PERMANOVA for colony membership in six pup colonies

```
# set seed to reproduce the same outcome
# (can vary due to different
# permutations!)
set.seed(123)

# set counter for while loop
perm_count <- c(99)

# iterate different significance levels
# with a while-loop end while-loop after
# the run for 99999 permutations
while (perm_count <= 99999) {
    permanova_result_pupcols <- adonis(scent ~
```

```r
        age + colony + colony:family, data = scent_factors,
        method = "bray", permutations = perm_count)
    print(permanova_result_pupcols)

    perm_count <- (perm_count * 10) + 9  # ends while-iteration after it increases to 999999
}  #while
```

```
##
## Call:
## adonis(formula = scent ~ age + colony + colony:family, data = scent_factors,      permutations = per
##
## Permutation: free
## Number of permutations: 99
##
## Terms added sequentially (first to last)
##
##                Df SumsOfSqs MeanSqs F.Model      R2 Pr(>F)
## colony          5    3.1874 0.63749  5.1748 0.19128   0.01 **
## colony:family   6    1.4037 0.23395  1.8991 0.08424   0.01 **
## Residuals      98   12.0727 0.12319         0.72449
## Total         109   16.6639                 1.00000
## ---
## Signif. codes:  0 '***' 0.001 '**' 0.01 '*' 0.05 '.' 0.1 ' ' 1
##
## Call:
## adonis(formula = scent ~ age + colony + colony:family, data = scent_factors,      permutations = per
##
## Permutation: free
## Number of permutations: 999
##
## Terms added sequentially (first to last)
##
##                Df SumsOfSqs MeanSqs F.Model      R2 Pr(>F)
## colony          5    3.1874 0.63749  5.1748 0.19128  0.001 ***
## colony:family   6    1.4037 0.23395  1.8991 0.08424  0.001 ***
## Residuals      98   12.0727 0.12319         0.72449
## Total         109   16.6639                 1.00000
## ---
## Signif. codes:  0 '***' 0.001 '**' 0.01 '*' 0.05 '.' 0.1 ' ' 1
##
## Call:
## adonis(formula = scent ~ age + colony + colony:family, data = scent_factors,      permutations = per
##
## Permutation: free
## Number of permutations: 9999
##
## Terms added sequentially (first to last)
##
##                Df SumsOfSqs MeanSqs F.Model      R2 Pr(>F)
## colony          5    3.1874 0.63749  5.1748 0.19128  1e-04 ***
## colony:family   6    1.4037 0.23395  1.8991 0.08424  2e-04 ***
## Residuals      98   12.0727 0.12319         0.72449
## Total         109   16.6639                 1.00000
## ---
```

```
## Signif. codes:  0 '***' 0.001 '**' 0.01 '*' 0.05 '.' 0.1 ' ' 1
##
## Call:
## adonis(formula = scent ~ age + colony + colony:family, data = scent_factors,      permutations = per
##
## Permutation: free
## Number of permutations: 99999
##
## Terms added sequentially (first to last)
##
##                Df SumsOfSqs MeanSqs F.Model      R2 Pr(>F)
## colony          5    3.1874 0.63749  5.1748 0.19128  1e-05 ***
## colony:family   6    1.4037 0.23395  1.8991 0.08424  6e-05 ***
## Residuals      98   12.0727 0.12319         0.72449
## Total         109   16.6639                 1.00000
## ---
## Signif. codes:  0 '***' 0.001 '**' 0.01 '*' 0.05 '.' 0.1 ' ' 1
```

Post-hoc tests for PERMANOVA results for six pup colonies

```
# pairwise PERMANOVA
pairwiseAdonis::pairwise.adonis(scent, scent_factors$colony,
    perm = 99999)
```

```
##                           pairs Df SumsOfSqs  F.Model         R2 p.value
## 1                    SSB vs FWB  1 0.8086332 6.080018 0.11038519 0.00001
## 2          SSB vs landing_beach  1 0.4880064 3.544865 0.08332062 0.00083
## 3               SSB vs main_bay  1 0.8584284 6.181387 0.13681269 0.00001
## 4           SSB vs natural_arch  1 0.5667911 4.172853 0.09665456 0.00010
## 5                SSB vs johnson  1 0.6168108 4.407128 0.10392422 0.00005
## 6          FWB vs landing_beach  1 0.5117674 4.168468 0.09885273 0.00009
## 7               FWB vs main_bay  1 0.9039828 7.289582 0.16095494 0.00001
## 8           FWB vs natural_arch  1 0.9556981 7.905832 0.17221847 0.00001
## 9                FWB vs johnson  1 0.8911846 7.145352 0.16185966 0.00001
## 10     landing_beach vs main_bay  1 0.4229462 3.322412 0.10607138 0.00186
## 11 landing_beach vs natural_arch  1 0.3694950 3.002564 0.09684889 0.00413
## 12      landing_beach vs johnson  1 0.3459312 2.694198 0.09073145 0.01225
## 13      main_bay vs natural_arch  1 0.7381617 5.917530 0.17446818 0.00001
## 14           main_bay vs johnson  1 0.4083747 3.137902 0.10411813 0.00024
## 15       natural_arch vs johnson  1 0.3053377 2.428242 0.08251399 0.01651
##    p.adjusted sig
## 1     0.00015  **
## 2     0.01245   .
## 3     0.00015  **
## 4     0.00150   *
## 5     0.00075  **
## 6     0.00135   *
## 7     0.00015  **
## 8     0.00015  **
## 9     0.00015  **
## 10    0.02790   .
## 11    0.06195
## 12    0.18375
## 13    0.00015  **
## 14    0.00360   *
```

```
## 15    0.24765
```

```r
# test for group dispersal
mod2 <- betadisper(vegdist(scent), scent_factors$colony,
    type = "median")
anova(mod2)
```

```
## Analysis of Variance Table
##
## Response: Distances
##            Df  Sum Sq   Mean Sq F value Pr(>F)
## Groups      5 0.02003 0.0040065   0.497 0.7779
## Residuals 104 0.83841 0.0080616
```

Re-evaluation of 2011 field season scent data

Perform non-metric multidimensional scaling

Re-evalution in PERMANOVA instead of ANOSIM

```r
## PERMANOVA
set.seed(123)
adonis(scent ~ age + colony + colony:family,
    data = peak_factors, permutations = 99999)
```

```
##
## Call:
## adonis(formula = scent ~ age + colony + colony:family, data = peak_factors,      permutations = 9999
##
## Permutation: free
## Number of permutations: 99999
##
## Terms added sequentially (first to last)
##
##               Df SumsOfSqs MeanSqs F.Model      R2 Pr(>F)
## age            1    0.2014 0.20143  0.9785 0.01013 0.4613
## colony         1    2.5430 2.54300 12.3538 0.12790  1e-05 ***
## colony:family  2    1.2880 0.64400  3.1285 0.06478  1e-05 ***
## Residuals     77   15.8503 0.20585         0.79719
## Total         81   19.8827                 1.00000
## ---
## Signif. codes:  0 '***' 0.001 '**' 0.01 '*' 0.05 '.' 0.1 ' ' 1
```

```r
# Test for heterogeneity
anova(betadisper(vegdist(scent), peak_factors$colony))
```

```
## Analysis of Variance Table
##
## Response: Distances
##           Df   Sum Sq   Mean Sq F value Pr(>F)
## Groups     1 0.000791 0.0007913   0.222 0.6388
## Residuals 80 0.285197 0.0035650
```

```r
anova(betadisper(vegdist(scent), peak_factors$age))
```

```
## Analysis of Variance Table
##
## Response: Distances
```

```
##           Df   Sum Sq    Mean Sq F value Pr(>F)
## Groups     1 0.000683 0.00068308  0.3488 0.5564
## Residuals 80 0.156659 0.00195824
```

```r
anova(betadisper(vegdist(scent), scent_nmds$BeachAge))
```

```
## Analysis of Variance Table
##
## Response: Distances
##           Df   Sum Sq    Mean Sq F value Pr(>F)
## Groups     3 0.001307 0.00043554  0.2134 0.8868
## Residuals 78 0.159188 0.00204087
```

Effect size estimate by PERMANOVA R^2 bootstrap

```r
## Load data and assign data to
## data.frames
load("RData/objects/R2_initial_season_btrap.RData")

old_season_colony <- paov_r2_results[[1]][[2]]
old_season_family <- paov_r2_results[[1]][[3]]

load("RData/objects/R2_replication_season_btrap.RData")
new_season_colony <- paov_r2_results[[1]][[2]]
new_season_family <- paov_r2_results[[1]][[3]]

MP_effectsize <- c(old_season_colony, new_season_colony,
    old_season_family, new_season_family)

MP_effectsize.groups <- c(rep("Colony S1",
    5000), rep("Colony S2", 5000), rep("Family S1",
    5000), rep("Family S2", 5000))

MP_effectsize.df <- data.frame(btrap_combined_results = MP_effectsize,
    btrap_subset_groups = MP_effectsize.groups)
```

Effect size estimate plot

```r
load("RData/objects/effect_size_df.RData")
# point estimates for PERMANOVA on
# non-bootstrapped (original) data
point_estimate <- c(0.1444734, 0.09168289,
    0.08780086, 0.1209394)
# point estimate groups for reasons of
# comprehensibility
point_estimate_groups <- c("Colony S1", "Colony S2",
    "Family S1", "Family S2")

# plot commands
MP_effectsize_gg <- ggplot(MP_effectsize.df,
    aes(y = btrap_combined_results, x = btrap_subset_groups,
        color = btrap_subset_groups)) + # this arranges the points according to
# their density
geom_quasirandom(alpha = 0.06, size = 3,
    width = 0.3, bandwidth = 1) + scale_color_manual(values = c("#E69F00",
```

```
    "#E69F00", "#CC79A7", "#CC79A7")) + # makes the boxplots
geom_boxplot(width = 0.35, outlier.shape = NA,
    color = "white", alpha = 0.1, lwd = 0.8) +
    annotate("point", x = 1, y = point_estimate[4],
        colour = "#000000", fill = "#CCCCCC",
        size = 2, shape = 21) + annotate("point",
    x = 2, y = point_estimate[3], colour = "#000000",
    fill = "#CCCCCC", size = 2, shape = 21) +
    annotate("point", x = 3, y = point_estimate[2],
        colour = "#000000", fill = "#CCCCCC",
        size = 2, shape = 21) + annotate("point",
    x = 4, y = point_estimate[1], colour = "#000000",
    fill = "#CCCCCC", size = 2, shape = 21) +
    # this is a possible theme of the plot,
# there are many others
theme_classic() + # changes the labels on the x axis
scale_y_continuous(limits = c(-0.01, 0.25),
    breaks = seq(0, 0.25, 0.05)) + scale_x_discrete(labels = c(`Family S2` = "Mother-offspring similari
    `Family S1` = "Mother-offspring similarity\noriginal study",
    `Colony S2` = "Colony membership\nreplication study",
    `Colony S1` = "Colony membership\noriginal study"),
    limits = c("Family S2", "Family S1",
        "Colony S2", "Colony S1")) + # geom_hline(yintercept = 0, linetype =
# 'dashed') +
xlab("") + # label for y axis
ylab("Explained variation [R²]") + # flips plot so everything is horizontal
coord_flip() + # adjust theme specifics
theme(panel.background = element_rect(colour = "black",
    size = 1.25, fill = NA), text = element_text(size = 15),
    axis.text = element_text(colour = "black"),
    legend.position = "none")


MP_effectsize_gg
```

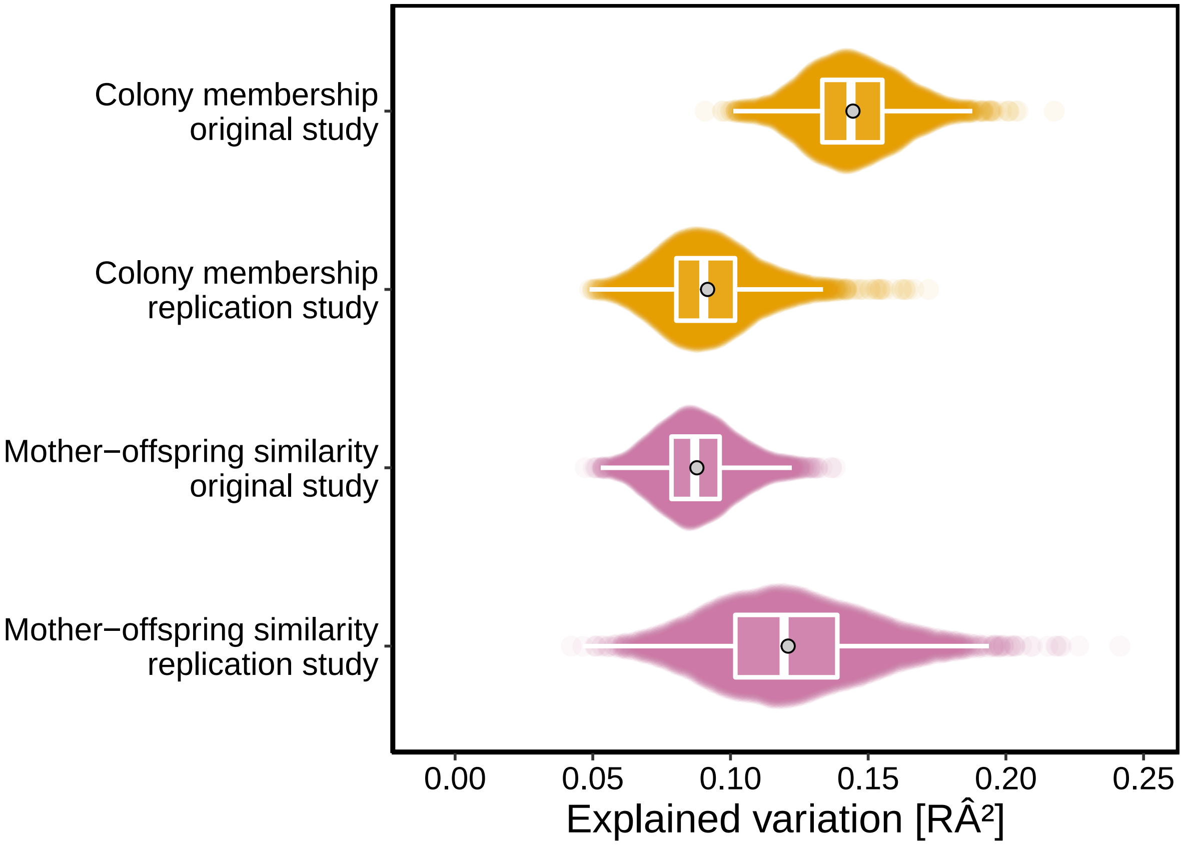

R2 Bootstrap Code

```
## creates function
## 'scent_btrap_r2_swarm_data' that
## performs bootstrap

# Bootstrap to track R2 values for
# randomized subsets. In addition,
# bootstrap cannot only be used to
# randomize the chemical data frame to
# evaluate R2 distribution as effect size
# estimates, but also to evaluate R2
# change for different subsets based on
# different premises. 1) Frequent peaks
# 2) Strong concentrations 3) Peaks
# identified by SIMPER

require(vegan)

# path: file path to
# scent_nmds-mompup2017_ssbfwb.RData',
# objects: scent_nmds, scent_nmds.obj,
# scent_factors, scent df.permutations:
# number of times the scent.df from
# loaded data will be permuted
```

```r
# nmds.permutations: number of
# permutation in nMDS using Bray-Curtis
# btrap.iterations: number of procedure
# repeats

scent_btrap_r2_swarm_data <- function(path,
    df.permutations = 15, nmds.permutations = 999,
    btrap.iterations = 5000) {
    # Create a data frame by permuting the
    # data for scent compounds data and also
    # ensure that each population*age occur
    # same amounts of time in the permutation
    # data frame.
    #---------------------------------------

    # load data frame with data of aligned
    # fur seal chromatograms
    load(path)
    scent_factors <- peak_factors
    # transfer BeachAge Column from
    # scent_nmds to meta data.frame
    # scent_factors
    scent_factors <- cbind(scent_factors,
        BeachAge = scent_nmds$BeachAge)

    # create index column for meta data frame
    scent_factors <- cbind(scent_factors,
        SampleIndex = 1:length(rownames(scent_factors)))


    # create data.frame to track PERMANOVA
    # results over repeated tests
    nonsubset_results_paov <- data.frame(R2_age = double(),
        p_colfam = double(), R2_residual = double(),
        F_Het = double(), p_Het = double())
    promcomp_results_paov <- data.frame(R2_age = double(),
        p_colfam = double(), R2_residual = double(),
        F_Het = double(), p_Het = double())
    highcomp_results_paov <- data.frame(R2_age = double(),
        p_colfam = double(), R2_residual = double(),
        F_Het = double(), p_Het = double())
    simper_results_paov <- data.frame(R2_age = double(),
        p_colfam = double(), R2_residual = double(),
        F_Het = double(), p_Het = double())

    # create list to store created objects in
    # an iteration
    iter_object_container <- list()

    for (i in 1:btrap.iterations) {


        # create data.frame subsets (colony
```

```r
# subset) by indexing the meta data.frame
scent.f.ssb.m <- scent_factors[scent_factors$BeachAge ==
    "SSB_1", ]
scent.f.fwb.m <- scent_factors[scent_factors$BeachAge ==
    "FWB_1", ]
scent.f.ssb.p <- scent_factors[scent_factors$BeachAge ==
    "SSB_2", ]
scent.f.fwb.p <- scent_factors[scent_factors$BeachAge ==
    "FWB_2", ]

# int vector of row index number of
# permuted scent.ssb data.frame row
# numbers will be used to create a
# permuted data.frame of evenly
# distributed draws of individuals
permute_rows_ssb_m <- sample(scent.f.ssb.m$SampleIndex,
    df.permutations, replace = T)
permute_rows_fwb_m <- sample(scent.f.fwb.m$SampleIndex,
    df.permutations, replace = T)
permute_rows_ssb_p <- sample(scent.f.ssb.p$SampleIndex,
    df.permutations, replace = T)
permute_rows_fwb_p <- sample(scent.f.fwb.p$SampleIndex,
    df.permutations, replace = T)

# create overall index number that can be
# used to index data.frame(scent): index
# corresponds to correct individual
perm_index_all <- c(permute_rows_ssb_m,
    permute_rows_fwb_m, permute_rows_ssb_p,
    permute_rows_fwb_p)

# create new data.frame with indeces
# found in permutation results vector
# perm_index_all
scent.permute <- scent[perm_index_all,
    ]
scent_factors.permute <- scent_factors[perm_index_all,
    ]
# rownames(scent.permute) ==
# rownames(scent_factors.permute) # TRUE
#---------------------------------------

# Perform analysis to find 3 subsets
# based on different premises with the
# permuted data frame.  Track 15 best
# performing compounds of an analysis
#---------------------------------------

## NDMS scale results count number of
## peaks that are not 0 per column
peak_count <- as.vector(apply(scent.permute,
    2, function(x) length(x[x > 0])))
```

```r
## add peaks in a column that are not 0 to
## estimate highest concentration peak sum
peak_add <- as.vector(apply(scent.permute,
    2, function(x) sum(x)))

## create dataframe with same name
## properties as scent.RData
compound_subset <- data.frame(name = colnames(scent.permute),
    peak_count, peak_add)

## sort data frame for most prominent
## compounds over all samples
most_abundant <- compound_subset %>%
    arrange(desc(peak_count))

## shorten scent matrix to only the 15
## most abundant compounds
scent.promcomp <- scent.permute[colnames(scent.permute) %in%
    most_abundant$name[1:15]]

## sort data frame for most highly
## concentrated compounds over all samples
most_concentration <- compound_subset %>%
    arrange(desc(peak_add))

## shorten scent matrix to only the 15
## most abundant compounds
scent.highcomp <- scent.permute[colnames(scent.permute) %in%
    most_concentration$name[1:15]]

## simper simper analysis and results
## array
sim <- with(scent_factors.permute,
    simper(scent.permute, colony))
best.compounds.simper.btrap <- summary(sim)[[1]]
# filter 15 compounds that contribute
# most towards dissimilarity of
# individuals
simper_comps <- as.numeric(rownames(best.compounds.simper.btrap))
best_comps <- simper_comps[1:15]
# subset peak data matrix {scent}
scent.simper.btrap <- scent.permute[,
    which(colnames(scent.permute) %in%
        as.character(best_comps))]
#---------------------------------------

# Take 15 identified compounds and limit
# nMDS of the permuted data frame
# (scent.permute) to only those compounds

#---------------------------------------

# bray-curtis similarity
```

```r
scent_nmds_regular.obj <- vegan::metaMDS(comm = scent.permute,
    k = 2, try = df.permutations,
    distance = "bray")
scent_nmds_count.obj <- vegan::metaMDS(comm = scent.promcomp,
    k = 2, try = df.permutations,
    distance = "bray")
scent_nmds_add.obj <- vegan::metaMDS(comm = scent.highcomp,
    k = 2, try = df.permutations,
    distance = "bray")
scent_nmds_simper.obj <- vegan::metaMDS(comm = scent.simper.btrap,
    k = 2, try = df.permutations,
    distance = "bray")

## get x and y coordinates
scent_nmds_regular <- as.data.frame(scent_nmds_regular.obj[["points"]])
scent_nmds_count <- as.data.frame(scent_nmds_count.obj[["points"]])
scent_nmds_add <- as.data.frame(scent_nmds_add.obj[["points"]])
scent_nmds_simper <- as.data.frame(scent_nmds_simper.obj[["points"]])

## add the colony as a factor to each
## sample
scent_nmds <- data.frame(MDS1r = scent_nmds_regular[["MDS1"]],
    MDS2r = scent_nmds_regular[["MDS2"]],
    MDS1c = scent_nmds_count[["MDS1"]],
    MDS2c = scent_nmds_count[["MDS2"]],
    MDS1a = scent_nmds_add[["MDS1"]],
    MDS2a = scent_nmds_add[["MDS2"]],
    MDS1s = scent_nmds_simper[["MDS1"]],
    MDS2s = scent_nmds_simper[["MDS2"]],
    age = scent_factors.permute[["age"]],
    colony = scent_factors.permute[["colony"]],
    family = scent_factors.permute[["family"]],
    BeachAge = scent_factors.permute[["BeachAge"]])
#----------------------------------------

# Perform PERMANOVA on distance matrix
# based limited scent compounds data
#----------------------------------------

# not subsetted
nonsubset.df_permanova <- adonis(scent.permute ~
    age + colony + colony:family,
    data = scent_factors.permute,
    permutations = 9999)
nonsubset.df_hetgeneity <- anova(betadisper(vegdist(scent.permute),
    scent_factors.permute$colony))

# track important values of statistical
# analysis in this run
nonsubset_iter_res_paov <- cbind(R2_age = nonsubset.df_permanova$aov.tab$R2[1],
    R2_colony = nonsubset.df_permanova$aov.tab$R2[2],
    R2_famcol = nonsubset.df_permanova$aov.tab$R2[3],
    R2_residual = nonsubset.df_permanova$aov.tab$R2[4],
```

```r
    F_Het = nonsubset.df_hetgeneity$`F value`[1],
    p_Het = nonsubset.df_hetgeneity$`Pr(>F)`[1])

# bind run values to track changes over
# iterations in the for-loop
nonsubset_results_paov <- rbind(nonsubset_results_paov,
    nonsubset_iter_res_paov)

# prom comps
promcomp.df_permanova <- adonis(scent.promcomp ~
    age + colony + colony:family,
    data = scent_factors.permute,
    permutations = 9999)
promcomp.df_hetgeneity <- anova(betadisper(vegdist(scent.promcomp),
    scent_factors.permute$colony))

promcomp_iter_res_paov <- cbind(R2_age = promcomp.df_permanova$aov.tab$R2[1],
    R2_colony = promcomp.df_permanova$aov.tab$R2[2],
    R2_famcol = promcomp.df_permanova$aov.tab$R2[3],
    R2_residual = promcomp.df_permanova$aov.tab$R2[4],
    F_Het = promcomp.df_hetgeneity$`F value`[1],
    p_Het = promcomp.df_hetgeneity$`Pr(>F)`[1])

promcomp_results_paov <- rbind(promcomp_results_paov,
    promcomp_iter_res_paov)

# high comps
highcomp.df_permanova <- adonis(scent.highcomp ~
    age + colony + colony:family,
    data = scent_factors.permute,
    permutations = 9999)
highcomp.df_hetgeneity <- anova(betadisper(vegdist(scent.highcomp),
    scent_factors.permute$colony))

highcomp_iter_res_paov <- cbind(R2_age = highcomp.df_permanova$aov.tab$R2[1],
    R2_colony = highcomp.df_permanova$aov.tab$R2[2],
    R2_famcol = highcomp.df_permanova$aov.tab$R2[3],
    R2_residual = highcomp.df_permanova$aov.tab$R2[4],
    F_Het = highcomp.df_hetgeneity$`F value`[1],
    p_Het = highcomp.df_hetgeneity$`Pr(>F)`[1])

highcomp_results_paov <- rbind(highcomp_results_paov,
    highcomp_iter_res_paov)
# SIMPER
simper.df_permanova <- adonis(scent.simper.btrap ~
    age + colony + colony:family,
    data = scent_factors.permute,
    permutations = 9999)
simper.df_hetgeneity <- anova(betadisper(vegdist(scent.simper.btrap),
    scent_factors.permute$colony))

simper_iter_res_paov <- cbind(R2_age = simper.df_permanova$aov.tab$R2[1],
    R2_colony = simper.df_permanova$aov.tab$R2[2],
```

```
        R2_famcol = simper.df_permanova$aov.tab$R2[3],
        R2_residual = simper.df_permanova$aov.tab$R2[4],
        F_Het = simper.df_hetgeneity$`F value`[1],
        p_Het = simper.df_hetgeneity$`Pr(>F)`[1])

    simper_results_paov <- rbind(simper_results_paov,
        simper_iter_res_paov)
    #----------------------------------------


    # # pack all this in a list to be later
    # on stored in a list that can be saved
    # again create name giving the iteration
    # step
    iteration_count <- paste0("iter_",
        i)

    # create list that stores relevant
    # workspace elements for an iteration
    # step
    iter_objects <- list(scent.permute = scent.permute,
        scent_factors.permute = scent_factors.permute,
        scent.promcomp = scent.promcomp,
        scent.highcomp = scent.highcomp,
        sim = sim, scent.simper.btrap = scent.simper.btrap,
        scent_nmds_regular.obj = scent_nmds_regular.obj,
        scent_nmds_count.obj = scent_nmds_count.obj,
        scent_nmds_add.obj = scent_nmds_add.obj,
        scent_nmds_simper.obj = scent_nmds_simper.obj,
        scent_nmds_regular = scent_nmds_regular,
        scent_nmds_count = scent_nmds_count,
        scent_nmds_add = scent_nmds_add,
        scent_nmds_simper = scent_nmds_simper,
        promcomp.df_permanova = promcomp.df_permanova,
        promcomp.df_hetgeneity = promcomp.df_hetgeneity,
        highcomp.df_permanova = highcomp.df_permanova,
        highcomp.df_hetgeneity = highcomp.df_permanova,
        simper.df_permanova = simper.df_permanova,
        simper.df_hetgeneity = simper.df_hetgeneity)

    # save everything as a list in a
    # container list, that stores
    # information/elements of all iteration
    # steps
    iter_object_container[[i]] <- iter_objects
    names(iter_object_container)[i] <- iteration_count

}  # end i

paov_r2_results <- list(regular = nonsubset_results_paov,
    promcomp = promcomp_results_paov,
    highcomp = highcomp_results_paov,
    simper_res = simper_results_paov)
```

```
    return(list(paov_r2_results = paov_r2_results,
        iter_object_container = iter_object_container))
}  # end function
```

Session information

```
## R version 3.5.3 (2019-03-11)
## Platform: x86_64-w64-mingw32/x64 (64-bit)
## Running under: Windows 10 x64 (build 18363)
##
## Matrix products: default
##
## locale:
## [1] LC_COLLATE=English_Germany.1252  LC_CTYPE=English_Germany.1252
## [3] LC_MONETARY=English_Germany.1252 LC_NUMERIC=C
## [5] LC_TIME=English_Germany.1252
##
## attached base packages:
## [1] stats     graphics  grDevices utils     datasets  methods   base
##
## other attached packages:
##  [1] pairwiseAdonis_0.0.1 cluster_2.0.7-1      forcats_0.4.0
##  [4] stringr_1.4.0        dplyr_0.8.0.1        purrr_0.3.1
##  [7] tidyr_0.8.3          tibble_2.0.1         tidyverse_1.2.1
## [10] ggbeeswarm_0.6.0     ggplot2_3.1.1        readr_1.3.1
## [13] vegan_2.5-4          lattice_0.20-38      permute_0.9-5
## [16] GCalignR_1.0.2
##
## loaded via a namespace (and not attached):
##  [1] Rcpp_1.0.0       lubridate_1.7.4  assertthat_0.2.0 digest_0.6.18
##  [5] R6_2.4.0         cellranger_1.1.0 plyr_1.8.4       backports_1.1.3
##  [9] evaluate_0.13    httr_1.4.0       pillar_1.3.1     rlang_0.3.1
## [13] lazyeval_0.2.1   readxl_1.3.0     rstudioapi_0.9.0 Matrix_1.2-15
## [17] rmarkdown_2.1    labeling_0.3     splines_3.5.3    munsell_0.5.0
## [21] broom_0.5.1      compiler_3.5.3   vipor_0.4.5      modelr_0.1.4
## [25] xfun_0.5         pkgconfig_2.0.2  mgcv_1.8-27      htmltools_0.3.6
## [29] tidyselect_0.2.5 crayon_1.3.4     withr_2.1.2      MASS_7.3-51.1
## [33] grid_3.5.3       nlme_3.1-137     jsonlite_1.6     gtable_0.2.0
## [37] magrittr_1.5     formatR_1.7      scales_1.0.0     cli_1.0.1
## [41] stringi_1.3.1    xml2_1.2.0       generics_0.0.2   tools_3.5.3
## [45] glue_1.3.0       beeswarm_0.2.3   hms_0.4.2        parallel_3.5.3
## [49] yaml_2.2.0       colorspace_1.4-0 rvest_0.3.2      knitr_1.22
## [53] haven_2.1.0
```
